# Association of SMAD4 loss with drug resistance in clinical cancer patients: A systematic meta-analysis

**DOI:** 10.1371/journal.pone.0250634

**Published:** 2021-05-28

**Authors:** Wei Xu, Sau Har Lee, Fengjun Qiu, Li Zhou, Xiaoling Wang, Tingjie Ye, Xudong Hu

**Affiliations:** 1 School of Basic Medical Science, Shanghai University of Traditional Chinese Medicine, Shanghai, China; 2 School of Biosciences, Faculty of Health and Medical Sciences, Taylor’s University, Subang Jaya, Selangor, Malaysia; 3 iSoftStone Information Technology (Group) Co., Ltd, Beijing, China; University of Texas Health Science Center, UNITED STATES

## Abstract

**Background:**

Drug resistance frequently led to the failure of chemotherapy for malignant cancers, hence causing cancer relapse. Thus, understanding mechanism of drug resistance in cancer is vital to improve the treatment efficacy. Here, we aim to evaluate the association between SMAD4 expression and the drug resistance in cancers by performing a meta-analysis.

**Method:**

Relevant studies detecting SMAD4 expression in cancer patients treated with chemo-drugs up till December 2020 were systematically searched in four common scientific databases using selected keywords. The pooled hazard ratio (HR) was the ratio of hazard rate between SMAD4^neg^ population vs SMAD4^pos^ population. The HRs and risk ratios (RRs) with 95% confidence intervals (CIs) were used to explore the association between SMAD4 expression losses with drug resistance in cancers.

**Result:**

After an initial screening according to the inclusion and exclusion criteria, eleven studies were included in the meta-analysis. There were a total of 2092 patients from all the included studies in this analysis. Results obtained indicated that loss of SMAD4 expression was significantly correlated with drug resistance with pooled HRs (95% CI) of 1.23 (1.01–1.45), metastasis with pooled RRs (95% CI) of 1.10 (0.97–1.25) and recurrence with pooled RRs (95% CI) of 1.32 (1.06–1.64). In the subgroup analysis, cancer type, drug type, sample size and antibody brand did not affect the significance of association between loss of SMAD4 expression and drug resistance. In addition, there was no evidence of publication bias as suggested by Begg’s test.

**Conclusion:**

Findings from our meta-analysis demonstrated that loss of SMAD4 expression was correlated with drug resistance, metastasis and recurrence. Therefore, SMAD4 expression could be potentially used as a molecular marker for cancer resistance.

## Introduction

Drug resistance in cancers contributes to the failure of chemotherapy and the subsequent cancer relapse, finally causing the death of patients [1–3]. Development of drug resistance is often associated with multiple intrinsic and extrinsic factors of cancer cells [1, 4]. Among this, signaling pathways activation plays an important role, including EGFR, Ras/MAPK, PI3K/Akt, Notch, Wnt/β-catenin and TGFβ pathways [5]. Of these, TGFβ signaling pathway regulates multiple cellular processes, including cell proliferation, differentiation, apoptosis, and specification of developmental fate during embryogenesis as well as in mature tissues [6].

The pathway ligands, such as TGFβs, BMPs and activins, bind to the TGFβ receptors to phosphorylate SMAD2 and SMAD3, forming a SMAD2/SMAD3 complex that subsequently interacts with SMAD4 to form a trimer complex, then which is translocated into the nucleus to initiate the downstream target genes transcription [7]. During cancer progression, TGFβ initially functions as a tumor suppressor, but eventually adopting promoter roles during the malignant stage [7]. It was well known that activated TGFβ induces epithelial-to-mesenchymal transition (EMT) that is often associated with cancer metastasis. However, the function of TGFβ in activating SMADs for EMT was unsure. Some studies showed that SMAD2 promotes cancer metastasis, resulting in poor survival of patients [8, 9]. Contrarily, several other studies revealed that SMAD2 suppressed EMT and cancer metastasis [10–13]. These results hence indicated that the exact role of SMAD2 in cancer is puzzling. As for SMAD3 function in cancer, most studies unanimously shown that SMAD3 promotes cancer progression and metastasis. Apart from this, a study by Michiko *et al*. revealed that a high frequency of SMAD4 gene mutations existed in human colorectal cancers, thus showing that inactivation of SMAD4 is important during cancer progression [14]. This finding suggested the role of SMAD4 as a suppressor gene. It is further supported by another study by Ding *et al*. who demonstrated an inhibition of prostate cancer growth and progression in the presence of SMAD4 [15]. Additionally, Charles reported that when SMAD4 was present in tumor cells, TGFβ had induced lethal EMT. Meanwhile, TGFβ had resulted in EMT and promoted tumor progression when SMAD4 was absent in tumor cells [16, 17]. The above studies proved that SMAD4 acts as a suppressor in the EMT process and tumorigenicity.

In the clinical studies, most of the reports showed that SMAD4 loss was correlated with cancer malignancy and prognosis. There are also several systematic reviews that studied the clinicopathological significance of SMAD4 loss in various cancers [3, 18–20], which had revealed that loss of SMAD4 was indeed associated with poorer survival and hence, is a negative prognostic indicator in patients. In agreement with this, Panagiotis *et al*. also found that SMAD4 inactivation had promoted the cancer progression and drug resistance of colorectal cancer in both *in vitro* and *in vivo* settings [21, 22]. However, there are other studies suggesting that SMAD4 inactivation had no significant correlation with the sensitization of pancreatic cancer cells to cisplatin or drug resistance [23, 24]. Thus, we conducted a meta-analysis of eligible studies to investigate an association between the loss of SMAD4 expression and the resistance of cancer to chemotherapy in order to clarify the exact prognostic value of SMAD4 loss in drug resistance.

## Materials and methods

### Publication search strategy

Systematic review of several databases was conducted in December 2020 with no lower limitation set for date of publication. The potentially relevant publications were searched in several established databases, including PubMed, EMBASE, Cochrane library and ISI Web of Science. Medical subheading (MeSH) terms related to SMAD4 (or DPC4) in combination with words related to cancer (Cancer* or Adenocarcinoma* or Carcinoma* or Tumor*), chemotherapy (or Chemoradiation or chemo* or drug), as well as terms related to patient (or patient*) were used to retrieve eligible studies.

**Fig 1:**
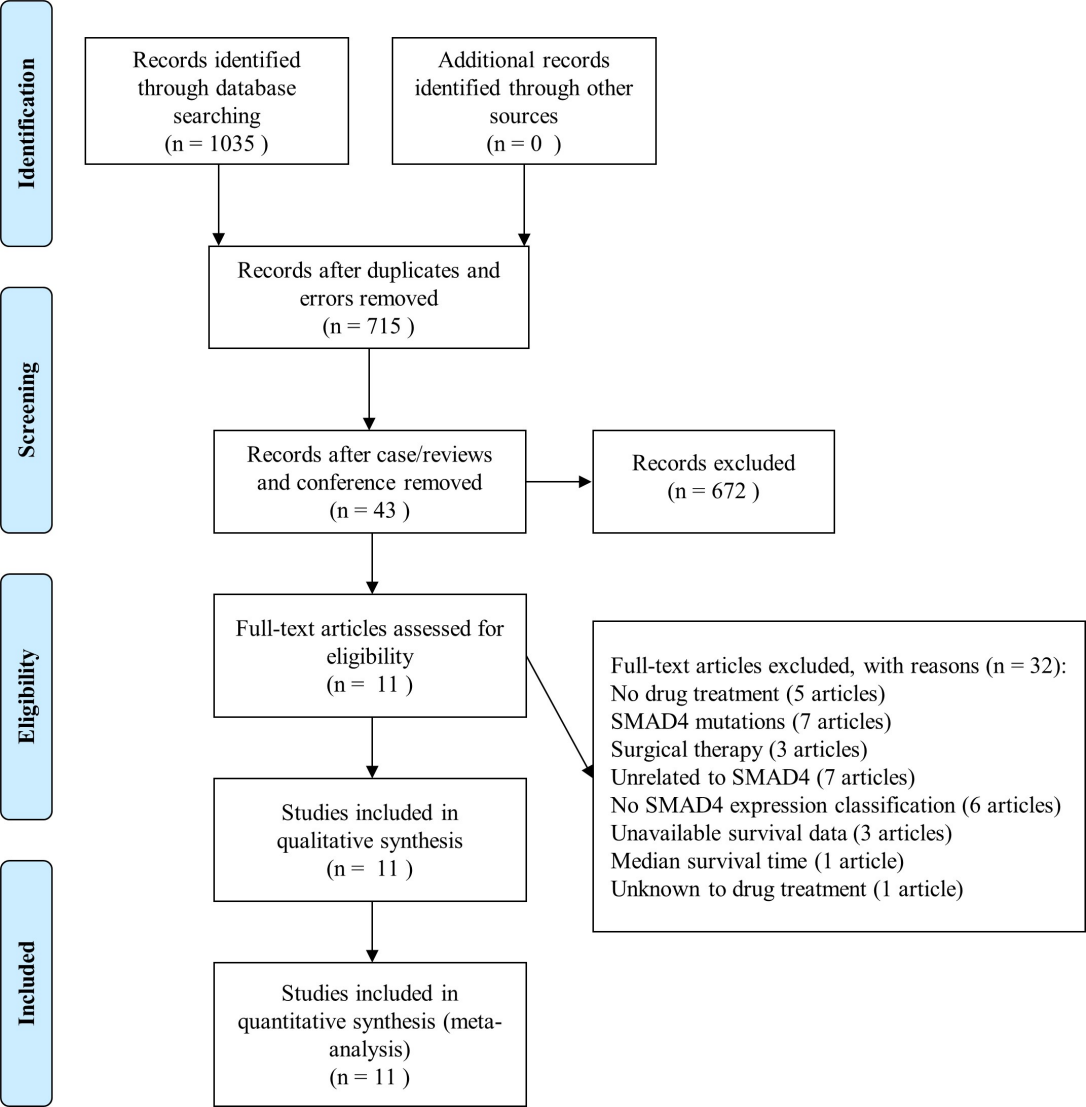
Methodological flow chart for the selection of papers in meta-analysis. (**Identification**) 1035 articles were identified from 4 online databases. (**Screening**) 992 records were excluded: 303 duplicated records and 17 error records; 111 cases/reviews and 258 conferences; 303 records unrelated to four key terms (patient*, cancer*/adeno*, chemo* and SMAD4/DPC4). (**Eligibility**) 32 records were excluded: 5 records did not include drug treatment in the SMAD4 group; 7 records involved SMAD4 mutation with unknown protein function; 3 records were under surgical therapy; 7 records were unrelated to SMAD4 expression in cancer progression and survival; 6 records were without SMAD4 expression classification in chemotherapy subgroup; 3 records were unavailable in survival data; 1 record only had median survival time without survival curve and 1 record was unknown for drug treatment. (**Included**) 11 studies were finally chosen for meta-analysis. n, the number of the selected articles.

1035 articles were extracted from the initial search using the combined MeSH terms. There were 152 articles identified from PubMed, 214 articles from ISI Web of Science, 652 articles from EMBASE and 17 articles from Cochrane library. A detailed screening process was illustrated as shown in Fig 1.

### Study selection criteria

Studies were included based on the following criteria: (1) the study objects were the patients; (2) the patients had to be treated with chemotherapy or chemoradiation; (3) the studies had to detect the SMAD4 expression level in tumor tissue; (4) the results in the studies had to show the overall survival curve data or present the HRs and 95% CIs. Studies were excluded according to the following criteria: (1) studies with duplicated data or a repeated analysis; (2) letters, reviews, case reports or conference; (3) study objects were xenografted animals with patient-derived cancer cells. (4) SMAD4 mutations with unknown protein function.

### Data extraction

The articles that have fulfilled both inclusion and exclusion criteria were reviewed and vital information was extracted by two investigators (X.W. and Lee S.H.) independently. Any disagreement was discussed and a consensus was reached for all issues. The following information were collected from each study: first author’s name, year of publication, cancer type, drug type, SMAD4 test, antibody brand, antibody dilution, sample size, number of SMAD4^pos^, number of SMAD4^neg^, outcome (overall survival), *P* value, HRs (the survival time of SMAD4^neg^ population vs SMAD4^pos^ population) with 95% CIs from multivariate analysis.

### Quality assessment

The quality of the included studies in meta-analysis was evaluated using the Newcastle-Ottawa quality assessment scale (NOS) [25]. The scale includes eight items with three different dimensions: selections (four items, one star for each item), comparability (one item, two stars), and outcome (three items, one star for each item). Total item stars were applied to quantitatively asses study quality. A higher star meant higher quality. Inconsistencies during scoring process by the two independent researchers were discussed to reach a consensus agreement.

### Statistical analysis

The pooled HRs were determined using HRs with their 95% CIs obtained from the studies. When the HR data cannot be obtained in the articles directly, a mathematical estimation based on the overall survival curve was performed according to the previously published methods demonstrated by Tierney *et al*. [26–28]. The pooled HR and 95% CI were used to estimate the effect of SMAD4 expression loss on the drug resistance of the cancers. A pooled HR > 1 implies that SMAD4-negative patients are resistant to the chemotherapy. The heterogeneity among studies was estimated using Cochran’s Q test (*P*_*heter*_ <0.05, significant heterogeneity) and the *I*^*2*^ statistic (*I*^*2*^ ≤ 50%, no or moderate heterogeneity; *I*^*2*^ >50%, strong heterogeneity). The random-effects model was applied in pooling the HRs (95% CIs) to avoid significant heterogeneity (*P*_*heter*_ <0.05 and *I*^*2*^ > 50%). A sensitivity analysis evaluating the stability of the results was performed by eliminating one study at a time. Publication bias was evaluated using the funnel analysis and begg’s test, with *P* < 0.05 to be considered significant. All statistical analysis was performed using the STATA software, version 16.0 (STATA Corporation, College Station, TX, USA). Meta-analysis was performed in STATA 16.0 using the *metan* package. All *P* values were two tailed tests.

## Results

### Study characteristics and quality assessment

The main characteristics of these studies were tabulated in Table 1. There were 2,092 patients in the included elven studies. All studies supplied the survival time or HRs of the patients, who were divided into SMAD4^pos^ and SMAD4^neg^ subgroups and treated with drugs. Six out of the eleven articles studied colorectal cancer. The other articles were about pancreatic cancer as shown in Table 1. SMAD4 expression was evaluated by immunochemistry staining in ten of the studies, while only one study used the RT-qPCR to evaluate whether the SMAD4 deletion or not. Five of eleven studies used SMAD4 antibodies produced by Santa Cruz Biotechnology Inc (Dallas, TX) while three studies used SMAD4 antibody purchased from Abcam Biotechnology Inc (Cambridge, UK). The sample size of six studies is over 150. Only three studies presented information on the drug administration route and dose used. The quality of the included studies was assessed according to the NOS scale. Among 11 studies, nine scored 8, one scored 7 and one scored 6 as shown in Table 2. The result showed that all the included studies were of high quality.

**Table 1:** Main characteristics of included studies.

Author	Year	Cancer type	Drug type	Smad4 detection	Antibody brand	Dilution	Sample size	Smad4^pos^	Smad4^neg^	Outcome	Drug administration route	Dose
Bachet J.B.	2012	Pancreatic	gemcitabine	IHC	Santa Cruz	1–50	249	87	162	OS	N.A	N.A
Baraniskin A.	2011	Colorectal	5-FU +oxaliplatin	IHC	Santa Cruz	1–100	190	125	65	OS,DFS	on days 1, 8, 15, 22 every day 36	2000 mg/m2/22 hours and 50mg/m2/2 hours
Fang Y.	2020	Pancreatic	gemcitabine	IHC	Abcam	1–100	80	21	59	OS	on days 1,8,15 for 6 times	1,000 mg/m2
Su F.	2016	Colorectal	FULV or FOLFOX4	IHC	Santa Cruz	1–150	174	N.A	N.A	OS,DFS	N.A	N.A
Shin S.H.	2017	Pancreatic	5-FU	IHC	Abcam	1–100	641	165	476	OS.DFS	N.A	N.A
Kozak M.K.	2015	Colorectal	5-FU	IHC	Santa Cruz	1–200	46	33	13	OS, PFS	N.A	N.A
Boulay J.L.	2002	Colorectal	5-FU	qPCR	N.A	N.A	202	67	135	OS,DFS	infusion for 7days	500 mg/m2
Alhopuro P.	2005	Colorectal	5-FU	IHC	Santa Cruz	1–100	75	65	10	OS, DFS	N.A	N.A
Ormanns S.	2017	Pancreatic	Gemcitabine +fluopyrimidine	IHC	Atlas	1–300	143	51	92	OS, DFS	N.A	N.A
Wasserman I.	2018	Colorectal	5-FU	IHC	Abcam	1–200	191	169	22	RFS	N.A	N.A
Herman J. M.	2011	Pancreatic	5-FU or gemcitabine	IHC	N.A	N.A	101	N.A	N.A	OS	N.A	N.A

pos, positive; neg, negative.

**Table 2:** The statistical data and quality assessment of studies.

Author	Year	The hazard ratio			The risk ratio of recurrence			The risk ratio of metastasis			NOS scale (*)
		HR	Lower 95%CI	Higher 95% CI	RR	Lower 95%CI	Higher 95% CI	RR	Lower 95%CI	Higher 95% CI	
Bachet J.B. [29]	2012	0.85	0.49	1.46	1.06	0.81	1.38	N.A	N.A	N.A	8
Baraniskin A. [30]	2011	1.88	1.15	3.1	N.A	N.A	N.A	N.A	N.A	N.A	8
Fang Y. [31]	2020	2.39	1.55	3.69	N.A	N.A	N.A	1.19	0.58	2.45	8
Su F. [32]	2016	1.14	0.84	1.54	N.A	N.A	N.A	1.158	0.89	1.5	8
Shin S.H. [33]	2017	1.21	0.99	1.48	1.42	1.12	1.8	N.A	N.A	N.A	8
Kozak M.K. [34]	2015	4.85	1.96	12.03	N.A	N.A	N.A	N.A	N.A	N.A	8
Boulay J.L. [35]	2002	2.09	1.29	3.39	2.08	0.83	5.2	N.A	N.A	N.A	8
Alhopuro P. [22]	2005	3.4	1.2	9.62	N.A	N.A	N.A	N.A	N.A	N.A	8
Ormanns S. [36]	2017	1.088	0.751	1.576	N.A	N.A	N.A	N.A	N.A	N.A	8
Wasserman I. [24]	2018	1.14	0.62	2.1	1.54	0.97	2.45	1.215	0.92	1.61	7
Herman J. M. [37]	2011	1.05	0.69	1.598	N.A	N.A	N.A	N.A	N.A	N.A	6

HR, Hazard Ratio; CI, confidence interval; RR, Risk Ratio.

### Loss of SMAD4 expression was correlated to cancer resistance

**Fig 2:**
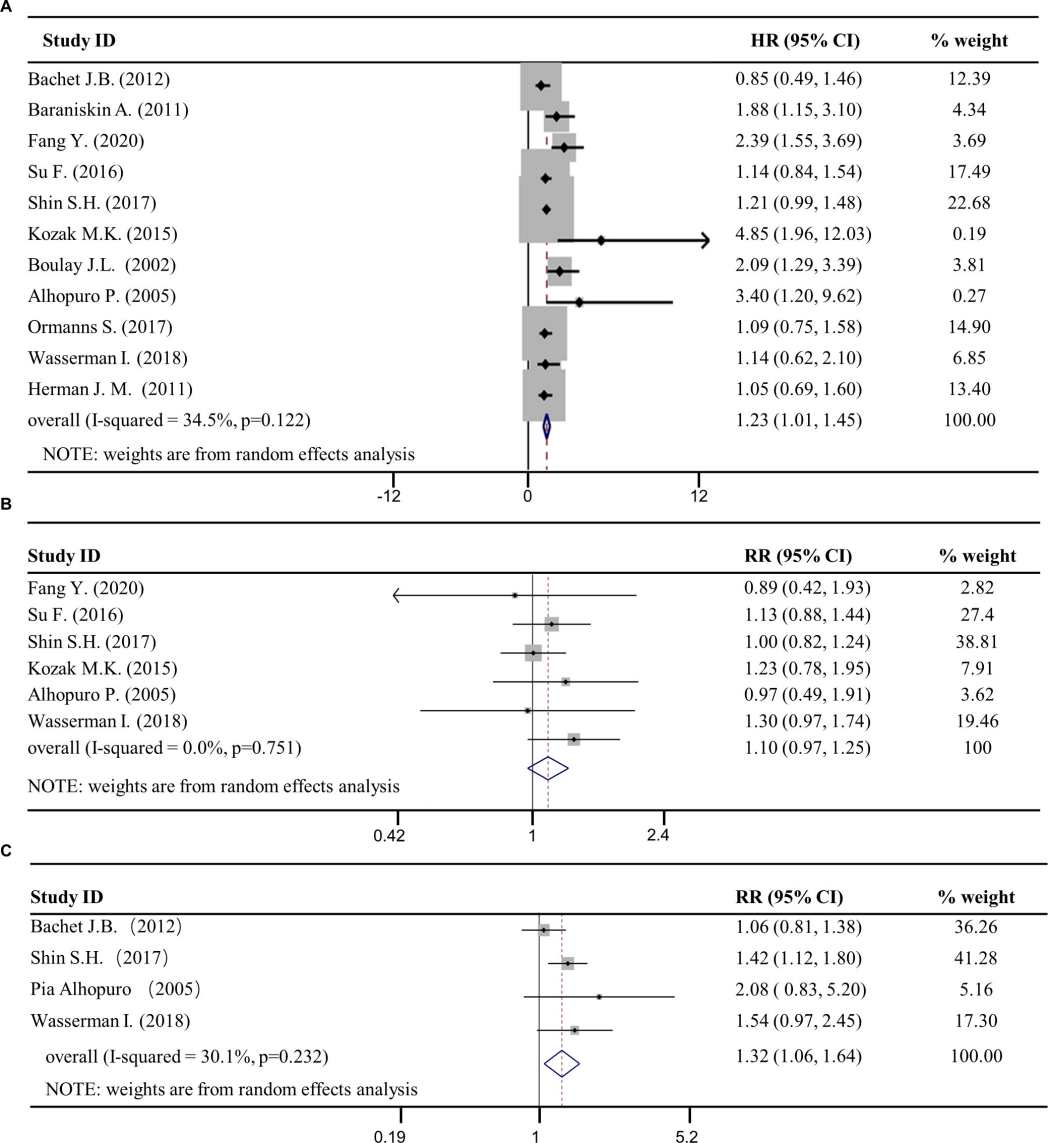
Forest plots of studies evaluating hazard ratios (HRs) and risk ratios (RR) of SMAD4 loss for resistance. Forest plots of 11 studies evaluating (**A**) HRs between SMAD4^neg^ and SMAD4^pos^ population, (**B**) RRs of metastasis between SMAD4^neg^ and SMAD4^pos^ population and (**C**) RRs of recurrence between SMAD4^neg^ and SMAD4^pos^ population. CI, confidence interval; HR, Hazard ratio.

Those eleven selected articles were subjected to multivariate analysis, whereby random-effects model was used to combine the effect of the SMAD4 expression loss on chemoresistance. The pooled HR (95% CI) was calculated to be 1.23 (1.01–1.45) (Fig 2A), indicating that the loss of SMAD4 expression in the cancers resulted in chemoresistance. Since metastasis and recurrence were also correlated to cancer resistance, we also pooled the RRs (risk ratio) of metastasis and recurrence in SMAD4 subpopulation to evaluate the effect of SMAD4 expression loss on chemoresistance. The pooled RRs (95% CI) were determined to be 1.10 (0.97, 1.25) and 1.32 (1.06, 1.64) (Fig 2B and 2C). Therefore, this result is indicating a significant correlation between SMAD4 expression loss in cancers with promotion of drug resistance.

**Fig 3:**
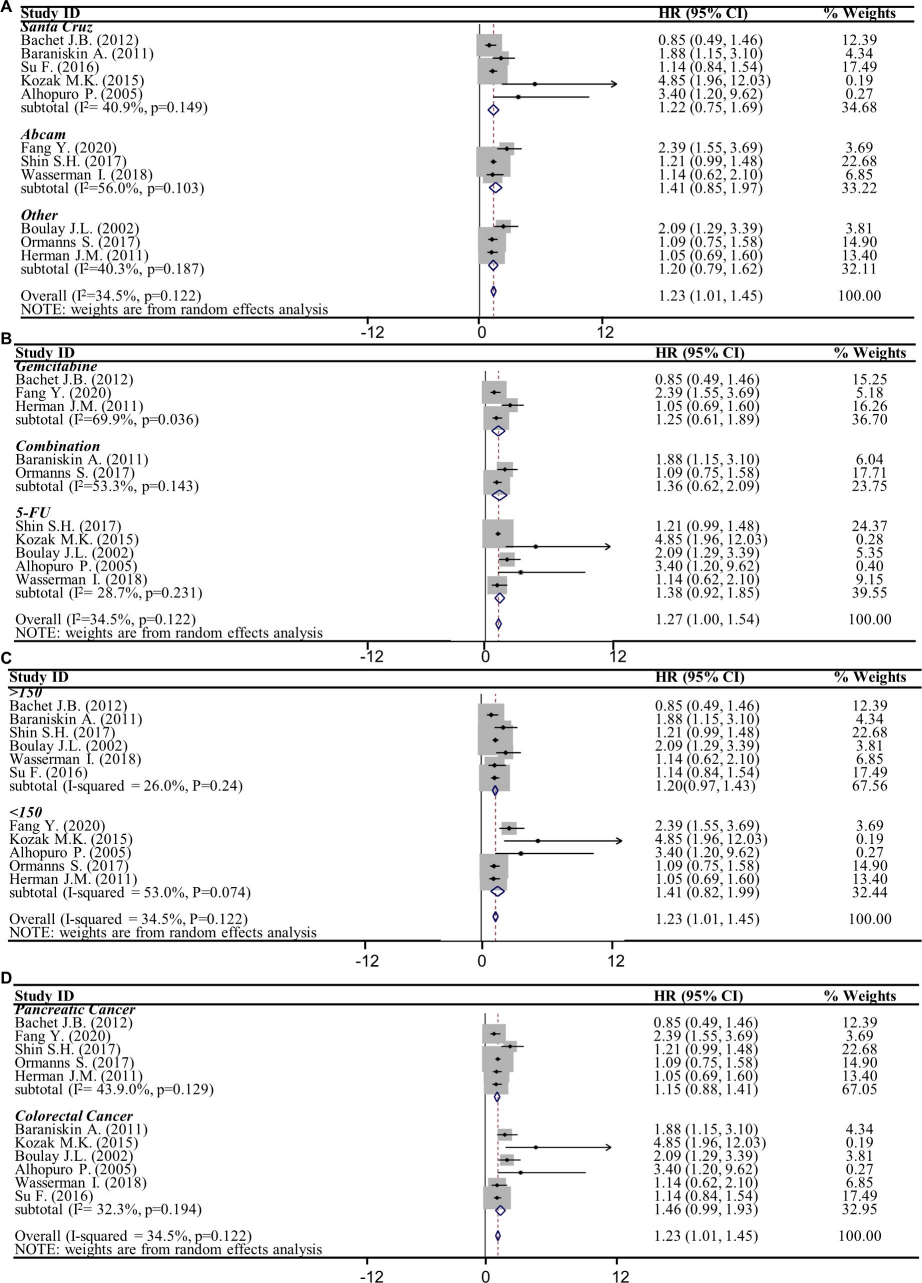
Forest plots of studies evaluating the association between SMAD4 loss to resistance and parameters by subgroup analysis. Forest plot of pooled HRs in (**A**) antibody brands subgroup, (**B**) drug types subgroup, (**C**) sample size subgroup and (**D**) cancer types subgroup between SMAD4^neg^ and SMAD4^pos^ population. CI, confidence interval; HR, Hazard ratio.

Subsequently, we analyzed the studies based on different cancer type’s subgroup. We found that the pooled HR (95% CI, *P*_ES_) was 1.15 (0.88–1.41, *<*0.001) in pancreatic cancer subgroup while the pooled HR (95% CI, *P*_ES_) was 1.46 (0.99–1.93, <0.001) in colorectal cancer subgroup (Fig 3D, Table 3). The result indicated that SMAD4 expression loss correlation with the drug resistance was independent on the cancer type.

**Table 3:** Stratified analysis of pooled HRs for cancer patients in different subgroups.

Variable	No. of studies	No. of Patients	HR (95% CI)	Heterogeneity			Model
				χ2	I^2^	P Value	
**Antibody type**							
Santa Cruz	5	734	1.22 (0.75, 1.69)	0.100	40.900%	0.149	random
Abcam	3	912	1.41 (0.85, 1.97)	0.138	56.000%	0.103	random
others	3	446	1.2 (0.79, 1.62)	0.054	40.300%	0.187	random
**Drug type**							
Gemcitabine	3	430	1.25 (0.61, 1.89)	0.211	69.900%	0.036	random
5-FU	5	1155	1.38 (0.92, 1.85)	0.083	28.700%	0.231	random
Combination	2	333	1.36 (0.62, 2.09)	0.168	53.500%	0.143	random
**Sample size**							
>150	6	1647	1.20 (0.97, 1.43)	0.0211	26.000%	0.24	random
<150	5	445	1.41 (0.82, 1.99)	0.177	53.000%	0.074	random
**Cancer type**							
Pancreatic cancer	5	1214	1.15 (0.88, 1.41)	0.038	43.900%	0.129	random
Colorectal cancer	6	878	1.46 (0.99, 1.93)	0.102	32.300%	0.194	random

No., number; HR, hazard ratio; CI, confidence interval.

Besides, we were also interested to know whether the correlation between SMAD4 loss and resistance is varied using different SMAD4 antibody brands. Therefore, the pooled HRs in the subgroup of SMAD4 antibody was analyzed. The results revealed the pooled HR (95% CI, *P*_ES_) in SANTA CRUZ subgroup was 1.22 (0.75–1.69, 0.004), pooled HR in Abcam subgroup was 1.41 (0.85–1.97, <0.001), while HR in the other brand subgroup was 1.20 (0.79–1.62, < 0.001) (Fig 3A, Table 3). The HR of each subgroup was >1 and *p* <0.01, which means SMAD4 expression loss significantly promoted cancer resistance independent on the antibody brand.

To exclude the possibility that drug resistance was varied in different type of chemo-drugs, we analyzed the pooled HR in drug type subgroup. It was found that HR (95% CI) of 5-FU chemo-drug subgroup was 1.38 (0.92–1.85), HR (95% CI) of gemcitabine subgroup was 1.25 (0.61–1.89), whereas HR (95% CI) of the combined drugs was 1.36 (0.62–2.09) (Fig 3B, Table 3). All the pooled HRs of the subgroup >1 and *P*_*ES*_ <0.01 (Table 3). This revealed that the cancer resistance induced by SMAD4 loss was not dependent on the type of chemo-drugs. Finally, we analyzed the sample size subgroup and found that the pooled HR (95% CI, *P*_ES_) was 1.20 (0.97–1.43, <0.01) when sample size greater than 150, the HRs was 1.41 (0.82–1.99, <0.01) when sample size less than 150 (Fig 3C, Table 3), which means SMAD4 expression loss correlation with resistance did not varied at different sample size in studies.

### Heterogeneity

The heterogeneity of pooled HRs in these 11 studies was tested and *I*^2^ value obtained was 34.5%, *P*_*heter*_ = 0.122, which revealed there was no heterogeneity among these eleven studies (Fig 2, Table 2). In the drug type subgroup analysis, the *I*^2^ value of 5-FU chemo-drug subgroup was 28.7%, and the *P*_*heter*_ >0.05. However, the *I*^2^ value of gemcitabine subgroup was 69.9% with *P*_*heter*_ <0.05, indicating that there was heterogeneity among these three studies of gemcitabine subgroups (Fig 3B, Table 3). For all others subgroups, the heterogeneity of Abcam subgroup, combination subgroup and >150 subgroup was greater than 50%, however, the *P*_*heter*_ was greater than 0.05 (Table 3). The heterogeneity of remaining subgroups was less than 50% and *P*_*heter*_ >0.05. The result indicated that there was no heterogeneity among the studies in these subgroups.

### Sensitivity analysis

**Fig 4:**
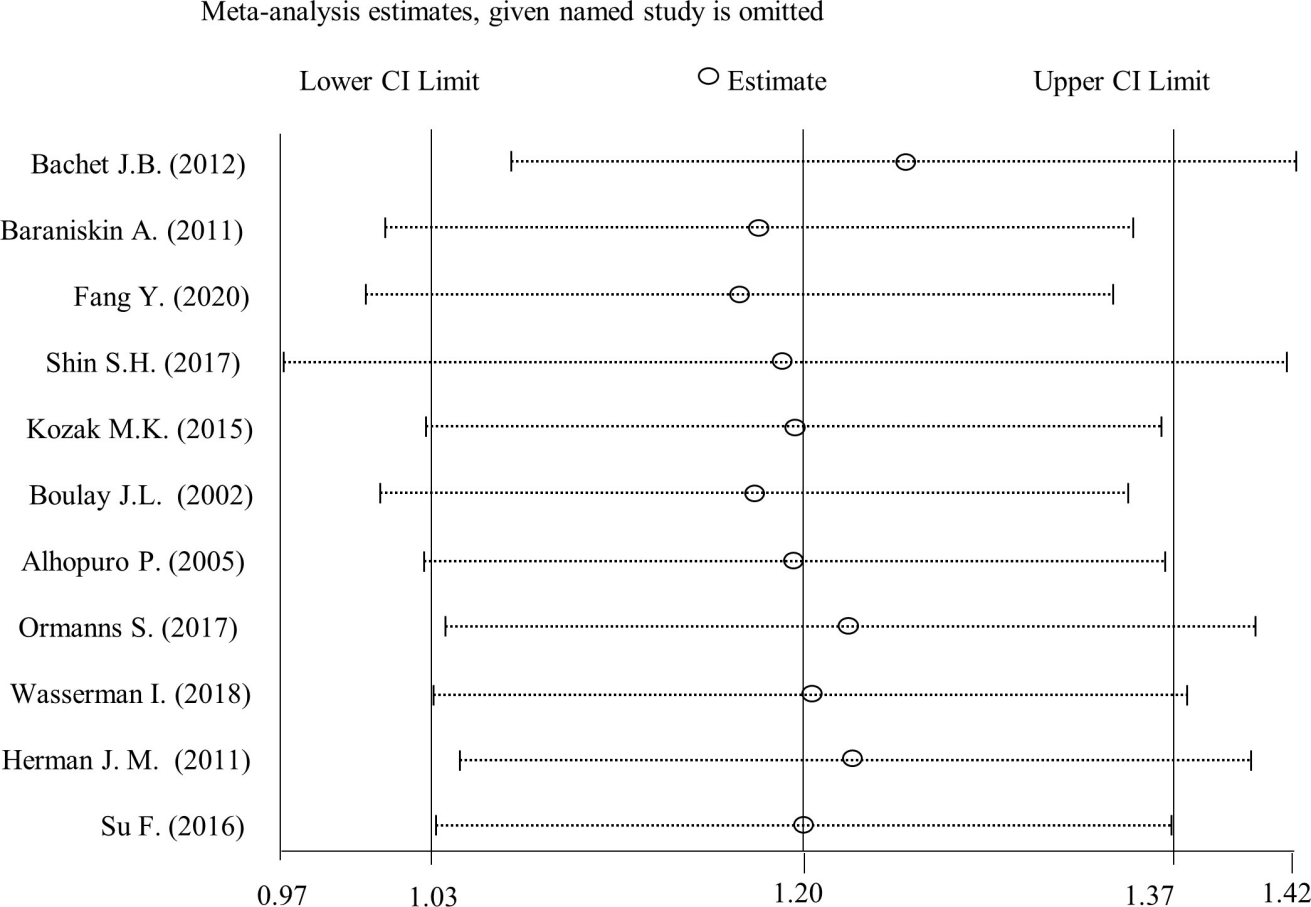
Effect of individual study on the pooled HRs for SMAD4 loss and drug resistance. X axis, the ranges of HRs; Y axis, the study ID.

To evaluate whether any single study had an influence on the pooled results, leave-one-out method was employed for sensitivity analysis. We sequentially removed each study and calculated the pooled HRs to evaluate the effect of an individual study on the pooled results. As shown in Fig 4, pooled HRs was stable after each study was removed sequentially, which suggested that any of these studies did not affect the pooled results significantly.

### Publication bias

**Fig 5:**
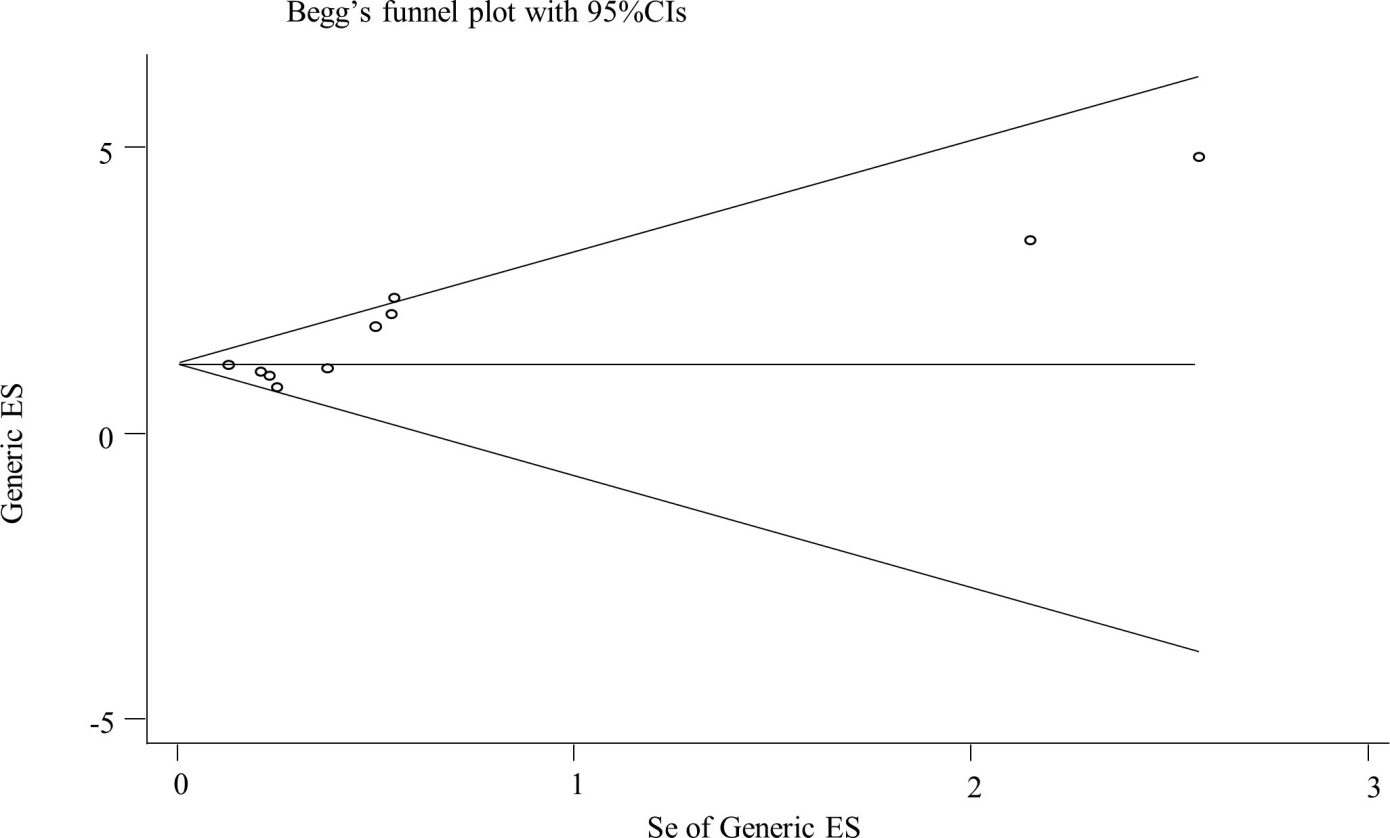
Begg’s funnel plot for publication bias assessment of all included studies. ES, effect size; se, standard error.

Begg’s funnel plot was used to evaluate publication bias, where no asymmetry was found in the plot (Fig 5). Meanwhile, the Begg’s test revealed the *P* value was 0.073 (Table 4), which indicated that there was no evidence for a significant publication bias in the meta-analysis.

**Table 4:** Begg’s test for funnel plot.

adj. Kendall’s Score (P-Q) = 23
Std. Dev. of Score = 12.5
Number of Studies = 11
z = 1.79
Pr > |z| = 0.073
z = 1.71 (continuity corrected)
Pr > |z| = 0.087 (continuity corrected)

## Discussion

SMAD4 is the downstream of TGFβ signaling and mutations associated with this gene has been reported in colorectal cancer [14, 38], head and neck carcinoma [39], seminoma germ cell tumors [40] and pancreatic cancers [41, 42]. The SMAD4 mutation resulted in gene inactivation that was correlated to tumorigenesis, metastasis, poor prognosis and radio-resistance. Although there were reports that analyzed the correlation of the SMAD4 expression loss with poorer cancer prognosis, it was still unclear whether SMAD4 expression loss was significantly related to drug resistance or not. This is the first study that used meta-analysis approach to prove that SMAD4 expression loss indeed promoted the cancers drug-resistance significantly.

So far, there are many reports showing that SMAD4 knock down had rendered the cancer cells resistant to the chemo-drugs in a xenograft model. Because the xenografts and patient data have vastly different biology and outcomes. Moreover, we cannot conclude that loss of SMAD4 expression could be potentially used as a molecular marker for cancer resistance in clinical therapy when we use animal xenograft models. Therefore, we excluded the animal xenograft models and selected patients with SMAD4 loss under chemo-drug treatment as our study focus. In our meta-analysis included 11 studies involving 2,092 patients, the sample size was enough to conduct the analysis for the association between SMAD4 expression loss and drug resistance. it is interesting to reveal that loss of SMAD4 expression was strongly associated with a worse prognosis for OS or RFS in the patients treated with chemo-drug. The result indicated SMAD4 expression loss resulted in the cancers drug-resistance significantly.

Although our findings proved that SMAD4 loss rendered the cancer cells resistance to chemotherapy, however, there are still some limitations in this study. This is because there may still exist certain degree of bias in this study since it is not possible to completely eliminate all potential bias. Firstly, the number of studies included in the meta-analysis was not enough. Besides, some of the HRs data were extracted using the strategies reported by Tierney *et al*. [28], hence the data calculated from the Kaplan-Meier curve may not be as precise as compared to obtaining data directly from the original article. Moreover, the SMAD4 expression level was determined only by immunochemistry and RT-qPCR. On top of that, the final antibody concentration from different brand may also be different. Therefore, the cutoff value of the result may be varied that could cause the bias. Based on these reasons, a random-effects model was adopted and subgroup analysis was performed to minimize the effect of this limitation factors.

## Supporting information

S1 Checklist PRISMA 2009 checklist for the manuscript. (PDF)

S1 Table The key information of the articles related to *SMAD4* mutation study. OS, overall survival; HR, Hazard Ratio.
(PDF)

## References

[1] Vasan N, Baselga J, Hyman DM. A view on drug resistance in cancer. Nature. 2019;575(7782):299–309. 10.1038/s41586-019-1730-1 31723286 PMC8008476

[2] Konieczkowski DJ, Johannessen CM, Garraway LA. A Convergence-Based Framework for Cancer Drug Resistance. Cancer cell. 2018;33(5):801–15. 10.1016/j.ccell.2018.03.025 29763622 PMC5957297

[3] Voorneveld PW, Jacobs RJ, Kodach LL, Hardwick JC. A Meta-Analysis of SMAD4 Immunohistochemistry as a Prognostic Marker in Colorectal Cancer. Translational oncology. 2015;8(1):18–24. 10.1016/j.tranon.2014.11.003 25749173 PMC4350636

[4] Holohan C, Van Schaeybroeck S, Longley DB, Johnston PG. Cancer drug resistance: an evolving paradigm. Nature reviews Cancer. 2013;13(10):714–26. 10.1038/nrc3599 24060863

[5] Panda M, Biswal BK. Cell signaling and cancer: a mechanistic insight into drug resistance. Molecular biology reports. 2019;46(5):5645–59. 10.1007/s11033-019-04958-6 31280421

[6] Shi Y, Massague J. Mechanisms of TGF-beta signaling from cell membrane to the nucleus. Cell. 2003;113(6):685–700. 10.1016/s0092-8674(03)00432-x 12809600

[7] Massague J. TGFbeta in Cancer. Cell. 2008;134(2):215–230. 10.1016/j.cell.2008.07.001 18662538 PMC3512574

[8] Shinto O, Yashiro M, Toyokawa T, Nishii T, Kaizaki R, Matsuzaki T, et al. Phosphorylated smad2 in advanced stage gastric carcinoma. BMC Cancer. 2010;10:652. 10.1186/1471-2407-10-652 21110833 PMC3001722

[9] Matsuzaki K, Kitano C, Murata M, Sekimoto G, Yoshida K, Uemura Y, et al. Smad2 and Smad3 phosphorylated at both linker and COOH-terminal regions transmit malignant TGF-beta signal in later stages of human colorectal cancer. Cancer Res. 2009;69(13):5321–30. 10.1158/0008-5472.CAN-08-4203 19531654

[10] Ju W, Ogawa A, Heyer J, Nierhof D, Yu L, Kucherlapati R, et al. Deletion of Smad2 in mouse liver reveals novel functions in hepatocyte growth and differentiation. Mol Cell Biol. 2006;26(2):654–67. 10.1128/MCB.26.2.654-667.2006 16382155 PMC1346892

[11] Hoot KE, Lighthall J, Han G, Lu SL, Li A, Ju W, et al. Keratinocyte-specific Smad2 ablation results in increased epithelial-mesenchymal transition during skin cancer formation and progression. Journal of Clinical Investigation. 2008;118(8):2722–32. 10.1172/JCI33713 18618014 PMC2447925

[12] Xu J, Lamouille S, Derynck R. TGF-beta-induced epithelial to mesenchymal transition. Cell research. 2009;19(2):156–72. 10.1038/cr.2009.5 19153598 PMC4720263

[13] Petersen M, Pardali E, van der Horst G, Cheung H, van den Hoogen C, van der Pluijm G, et al. Smad2 and Smad3 have opposing roles in breast cancer bone metastasis by differentially affecting tumor angiogenesis. Oncogene. 2010;29(9):1351–61. 10.1038/onc.2009.426 20010874

[14] Miyaki M, Iijima T, Konishi M, Sakai K, Ishii A, Yasuno M, et al. Higher frequency of Smad4 gene mutation in human colorectal cancer with distant metastasis. Oncogene. 1999;18(20):3098–103. 10.1038/sj.onc.1202642 10340381

[15] Ding Z, Wu CJ, Chu GC, Xiao Y, Ho D, Zhang J, et al. SMAD4-dependent barrier constrains prostate cancer growth and metastatic progression. Nature. 2011;470(7333):269–73. 10.1038/nature09677 21289624 PMC3753179

[16] David CJ, Huang YH, Chen M, Su J, Zou Y, Bardeesy N, et al. TGF-beta Tumor Suppression through a Lethal EMT. Cell. 2016;164(5):1015–30. 10.1016/j.cell.2016.01.009 26898331 PMC4801341

[17] Siraj AK, Pratheeshkumar P, Divya SP, Parvathareddy SK, Bu R, Masoodi T, et al. TGFbeta-induced SMAD4-dependent Apoptosis Proceeded by EMT in CRC. Molecular cancer therapeutics. 2019;18(7):1312–22. 10.1158/1535-7163.MCT-18-1378 31053577

[18] Wang JD, Jin K, Chen XY, Lv JQ, Ji KW. Clinicopathological significance of SMAD4 loss in pancreatic ductal adenocarcinomas: a systematic review and meta-analysis. Oncotarget. 2017;8(10):16704–11. 10.18632/oncotarget.14335 28053288 PMC5369995

[19] Du Y, Zhou X, Huang Z, Qiu T, Wang J, Zhu W, et al. Meta-analysis of the prognostic value of smad4 immunohistochemistry in various cancers. PLoS One. 2014;9(10):e110182. 10.1371/journal.pone.0110182 25333693 PMC4198206

[20] Shugang X, Hongfa Y, Jianpeng L, Xu Z, Jingqi F, Xiangxiang L, et al. Prognostic Value of SMAD4 in Pancreatic Cancer: A Meta-Analysis. Translational oncology. 2016;9(1):1–7. 10.1016/j.tranon.2015.11.007 26947875 PMC4800056

[21] Papageorgis P, Cheng KH, Ozturk S, Gong Y, Lambert AW, Abdolmaleky HM, et al. Smad4 Inactivation Promotes Malignancy and Drug Resistance of Colon Cancer. Cancer Research. 2011;71(3):998–1008. 10.1158/0008-5472.CAN-09-3269 21245094 PMC3075468

[22] Alhopuro P, Alazzouzi H, Sammalkorpi H, Davalos V, Salovaara R, Hemminki A, et al. SMAD4 levels and response to 5-fluorouracil in colorectal cancer. Clin Cancer Res. 2005;11(17):6311–6. 10.1158/1078-0432.CCR-05-0244 16144935

[23] Cui Y, Brosnan JA, Blackford AL, Sur S, Hruban RH, Kinzler KW, et al. Genetically defined subsets of human pancreatic cancer show unique in vitro chemosensitivity. Clin Cancer Res. 2012;18(23):6519–30. 10.1158/1078-0432.CCR-12-0827 22753594 PMC3513499

[24] Wasserman I, Lee LH, Ogino S, Marco MR. SMAD4 Loss in Colorectal Cancer Patients Correlates with Recurrence, Loss of Immune Infiltrate, and Chemoresistance. Clin Cancer Res. 2019;25(6):1948–56. 10.1158/1078-0432.CCR-18-1726 30587545 PMC6421131

[25] Stang A. Critical evaluation of the Newcastle-Ottawa scale for the assessment of the quality of nonrandomized studies in meta-analyses. European journal of epidemiology. 2010;25(9):603–5. 10.1007/s10654-010-9491-z 20652370

[26] Parmar MK, Torri V, Stewart L. Extracting summary statistics to perform meta-analyses of the published literature for survival endpoints. Statistics in medicine. 1998;17(24):2815–34. 10.1002/(sici)1097-0258(19981230)17:24&lt;2815::aid-sim110&gt;3.0.co;2-8 9921604

[27] Williamson PR, Smith CT, Hutton JL, Marson AG. Aggregate data meta-analysis with time-to-event outcomes. Statistics in medicine. 2002;21(22):3337–51. 10.1002/sim.1303 12407676

[28] Tierney JF, Stewart LA, Ghersi D, Burdett S, Sydes MR. Practical methods for incorporating summary time-to-event data into meta-analysis. Trials. 2007;8:16. 10.1186/1745-6215-8-16 17555582 PMC1920534

[29] Bachet JB, Maréchal R, Demetter P, Bonnetain F, Couvelard A, Svrcek M, et al. Contribution of CXCR4 and SMAD4 in predicting disease progression pattern and benefit from adjuvant chemotherapy in resected pancreatic adenocarcinoma. Annals of Oncology. 2012;23(9):2327–35. 10.1093/annonc/mdr617 22377565

[30] Baraniskin A, Munding J, Schulmann K, Meier D, Porschen R, Arkenau HT, et al. Prognostic Value of Reduced SMAD4 Expression in Patients With Metastatic Colorectal Cancer Under Oxaliplatin-Containing Chemotherapy: A Translational Study of the AIO Colorectal Study Group. Clinical Colorectal Cancer. 2011;10(1):24–9. 10.3816/CCC.2011.n.003 21609932

[31] Fang Y, Han X, Li JN, Kuang TT, Lou WH. HEATR1 Deficiency Promotes Chemoresistance via Upregulating ZNF185 and Downregulating SMAD4 in Pancreatic Cancer. Journal of Oncology. 2020;2020. 10.1155/2020/3181596 32565799 PMC7271247

[32] Su F, Li XM, You K, Chen MW, Xiao JB, Zhang YF, et al. Expression of VEGF-D, SMAD4, and SMAD7 and Their Relationship with Lymphangiogenesis and Prognosis in Colon Cancer. Journal of Gastrointestinal Surgery. 2016;20(12):2074–82. 10.1007/s11605-016-3294-9 27730400

[33] Shin SH, Kim HJ, Hwang DW, Lee JH, Song KB, Jun E, et al. The DPC4/SMAD4 genetic status determines recurrence patterns and treatment outcomes in resected pancreatic ductal adenocarcinoma: A prospective cohort study. Oncotarget. 2017;8(11):17945–59. 10.18632/oncotarget.14901 28160547 PMC5392299

[34] Kozak MM, von Eyben R, Pai J, Vossler SR, Limaye M, Jayachandran P, et al. Smad4 inactivation predicts for worse prognosis and response to fluorouracil-based treatment in colorectal cancer. Journal of Clinical Pathology. 2015;68(5):341–5. 10.1136/jclinpath-2014-202660 25681512

[35] Boulay JL, Mild G, Lowy A, Reuter J, Lagrange M, Terracciano L, et al. SMAD4 is a predictive marker for 5 fluorouracil based chemotherapy in patients with colorectal cancer. British journal of cancer. 2002;87(6):630–4. 10.1038/sj.bjc.6600511 12237773 PMC2364238

[36] Ormanns S, Haas M, Remold A, Kruger S, Holdenrieder S, Kirchner T, et al. The Impact of SMAD4 Loss on Outcome in Patients with Advanced Pancreatic Cancer Treated with Systemic Chemotherapy. International Journal of Molecular Sciences. 2017;18(5). 10.3390/ijms18051094 28534865 PMC5455003

[37] Herman JM, Hsu CC, Fishman EK, Hruban RH, Lin SH, Hacker-Prietz A, et al. Correlation of DPC4 status with outcomes in pancreatic adenocarcinoma patients receiving adjuvant chemoradiation. Journal of Clinical Oncology. 2011;29(4).

[38] Fleming NI, Jorissen RN, Mouradov D, Christie M, Sakthianandeswaren A, Palmieri M, et al. SMAD2, SMAD3 and SMAD4 mutations in colorectal cancer. Cancer Res. 2013;73(2):725–35. 10.1158/0008-5472.CAN-12-2706 23139211

[39] Lin LH, Chang KW, Cheng HW, Liu CJ. SMAD4 Somatic Mutations in Head and Neck Carcinoma Are Associated With Tumor Progression. Frontiers in oncology. 2019;9:1379. 10.3389/fonc.2019.01379 31867281 PMC6909744

[40] Bouras M, Tabone E, Bertholon J, Sommer P, Bouvier R, Droz JP, et al. A novel SMAD4 gene mutation in seminoma germ cell tumors. Cancer Res. 2000;60(4):922–8. 10706106

[41] Blackford A, Serrano OK, Wolfgang CL, Parmigiani G, Jones S, Zhang X, et al. SMAD4 gene mutations are associated with poor prognosis in pancreatic cancer. Clinical cancer research: an official journal of the American Association for Cancer Research. 2009;15(14):4674–9. 10.1158/1078-0432.CCR-09-0227 PMC2819274 19584151

[42] Wang F, Xia X, Yang C, Shen J, Mai J, Kim HC, et al. SMAD4 Gene Mutation Renders Pancreatic Cancer Resistance to Radiotherapy through Promotion of Autophagy. Clinical cancer research: an official journal of the American Association for Cancer Research. 2018;24(13):3176–85. 10.1158/1078-0432.CCR-17-3435 29602802 PMC6345154

