# Supplementary material for: Association of SMAD4 loss with drug resistance in clinical cancer patients: A systematic meta-analysis
Source: PLoS One. 2021 May 28;16(5):e0250634. doi: 10.1371/journal.pone.0250634 (PMC8162645; doi:10.1371/journal.pone.0250634)
Supplement: S1 Table — OS, overall survival; HR, Hazard Ratio. (PDF) [file pone.0250634.s002.pdf]

S1 Table. The key information of the articles related to SMAD4 mutation study.

OS, overall survival; HR, Hazard Ratio.

Author	Year	Mutation type	Mutation function	Chemo-therapy	Survival curve, OS, HR
Karuna G.	2016	various mutation in different patients	loss of function	No	yes
Takahiro Y.	2019	various mutation in different patients	unknown	No	No
George Z.	2019	various mutation in different patients	unknown	Yes	No
Dan J.	2019	various mutation in different patients	unknown	yes	yes
Ju Y.	2017	one SNP mutation in all patients	3 Prime UTR Variant	radiotherapy	yes
Arndt S.	2020	various SNV mutation in different patients	unknown	No	yes
Zhu M.	2018	various mutation in different patients	unknown	yes	yes
Salvatore P.	2017	various mutation in different patients	unknown	radiotherapy	No
